# Protocol for assessing feasibility, acceptability and fidelity of screening for antenatal depression (FAFSAD) by midwives in Blantyre District, Malawi

**DOI:** 10.1186/s40814-021-00775-6

**Published:** 2021-01-26

**Authors:** Genesis Chorwe-Sungani, Modesta Mwagomba, Kazione Kulisewa, Ellen Chirwa, Diana Jere, Jennifer Chipps

**Affiliations:** 1 Kamuzu College of Nursing, University of Malawi, Chipatala Avenue, P. O. Box 415, Blantyre, Malawi; 2 Blantyre District Health Office, Chipatala Avenue, P/Bag 66, Blantyre, Malawi; 3 College of Medicine, University of Malawi, Mahatma Ghandi Rd, P/Bag, 360 Blantyre, Malawi; 4 University of the Western Cape, Robert Sobukwe Rd, Bellville, Cape Town, 7535 South Africa

## Abstract

**Background:**

Depression is often underdiagnosed by treating health professionals. This is a situation in Malawi where there is no routine screening of depression at antenatal clinics. Recently, a Screening Protocol for Antenatal Depression (SPADe) that can be used by midwives to screen for antenatal depression was developed in Blantyre District. SPADe proposes multistage screening of antenatal depression by midwives which may enable early detection and treatment of pregnant women with depression. Proper treatment of antenatal depression can assist in achieving Sustainable Development Goals (SDGs). However, utilisation of SPADe in clinical practice to screening for depression in antenatal clinics has not been established yet. Therefore, the primary aim of this study is to assess feasibility of screening for depression by midwives using SPADe in antenatal clinics in Blantyre District. The secondary aim was to assess acceptability and fidelity of screening for depression by midwives using SPADe in antenatal clinics in Blantyre District.

**Methods:**

This will be a feasibility study which will consist of scientific investigations that will support movement of evidence-based, effective health care approach, SPADe, from the clinical knowledge base into routine use. This study will consist three phases: phase 1 will introduce SPADe in antenatal clinics in Blantyre District where screening of depression is almost none existent; phase 2 will implement screening of depression using SPADe in antenatal clinics in Blantyre District; and phase 3 will evaluate the screening of antenatal depression using SPADe to establish its feasibility, acceptability and fidelity in antenatal clinics in Blantyre District.

**Discussion:**

This study will establish and document feasibility, acceptability and fidelity of screening for depression by midwives using SPADe in antenatal clinics in Blantyre District. It is expected that midwives will develop more confidence in detecting and dealing with antenatal depression. Consequently, there will be increased numbers of pregnant women detected with depression by midwives and increased accessibility to mental health care by pregnant women in antenatal clinics.

**Supplementary Information:**

The online version contains supplementary material available at 10.1186/s40814-021-00775-6.

## Background

Depression is a serious health problem affecting pregnant women during all stages of their pregnancy [1]. In Malawi, major depressive disorders (10.7% to 25.8%) are prevalent among pregnant women [2, 3]. These figures fall within prevalence rates range of depressive disorders during pregnancy (8.3% to 41%) reported in sub-Saharan Africa [4]. Antenatal depression can lead to adverse effects on both maternal and foetal well-being [5, 6] and also result into the poor uptake of antenatal services [7]. Pregnant women with untreated depression have a higher chance of obstetric complications, premature deliveries and low birthweight infants [8]. Furthermore, antenatal depression is a risk factor for postnatal depression [9]. This is a cause for public health concern and necessitates proper screening of antenatal depression so that women with depressive symptoms are timely identified and treated. Screening for depression encompasses the use of instruments for measuring symptoms of depression to identify patients who may have depression but who have not sought treatment and whose depression has not already been detected by clinicians [10]. However, midwives do not routinely screen for antenatal depression considering that they have increased workload in local antenatal settings where screening for depression is not a key task. This is contrary to a duty of midwives which requires of them to screen pregnant women for various conditions [11] including depression during antenatal care.

Midwives are expected to routinely screen for depression in all pregnant women [12] to improve detection of antenatal depression [13]. The American College of Obstetricians and Gynaecologists recommended that pregnant women should be screened for antenatal depression using a standardised and validated instrument [14]. Screening for depression in antenatal clinics can be done in stages whereby various instruments are combined [15]. Screening in stages may involve a two-step process where a short screening instrument is used to identify potential cases and, for those who screen positive, a second, often more detailed instrument with greater specificity, is used to confirm the diagnosis [16]. This is in agreement with Chorwe-Sungani [17] who proposed that screening of depression in antenatal clinics in Blantyre District can be implemented using a Screening Protocol for Antenatal Depression (SPADe).

The SPADe recommends the use of a two-step process whereby the 3-item screener (ultra-brief instrument) should initially be used by midwives to identify potential depression cases followed by Self Reporting Questionnaire (SRQ) 20 to confirm the cases and later a gold standard administered by a mental health specialist to confirm a diagnosis [15]. This is supported by Whooley [18] who asserted that any positive screen result on an ultra-brief instrument must be followed by a clinical interview to confirm the presence of depression. Midwives can be able to screen for depression and refer pregnant women appropriately after receiving some training [19]. However, midwives may consider screening for antenatal depression to be too demanding and requiring too much effort, and this may result in a decreased frequency of screening [20]. Midwives may also have limited knowledge and skills for detecting and treating mental disorders [5] including antenatal depression.

Working from the premise that midwives may be trained to screen and refer antenatal depression cases in low-resource settings [19], it is important that midwives in local antenatal clinics are trained on the use of SPADe. This may help midwives to integrate screening of depression into antenatal services [21] and provide effective mental health care in an organised manner [22]. Nonetheless, what remains unknown is acceptability, feasibility and fidelity of screening for depression by midwives using SPADe in antenatal clinics in Blantyre District.

### Rationale for the research project

The Lancet series on maternal mental health have recognised the clinical and public health importance of antenatal depression [8, 23–25]. Prevalence of antenatal depression is high [23] and is linked with adverse child outcomes in low resource settings where pregnant women have increased exposure to risk factors of depression [26]. The adverse mental health outcomes for the child include an increased risk of anxiety, depression, attention deficit hyperactivity disorder and conduct disorder [26]. It is documented that pregnant women with untreated depression have a higher likelihood of obstetric complications, premature deliveries and low birthweight infants [8]. This may be the case in Malawi where obstetric complications are common and many babies with low birthweight are born.

Apart from the aforementioned adverse effects, antenatal depression and HIV infection form a vicious cycle, whereby the symptoms of each disease worsen the status of the other, and each needs to be sufficiently treated for the pregnant woman to become healthy [24]. It is of public health concern that pregnant women with co-morbidity of depression and HIV infection are less likely to adhere to antiretroviral therapy, which is critical for her survival and prevention of HIV transmission to the newborn [25]. Stringer and colleagues [24] recommended integration depression-screening technique in antenatal services that could identify a large proportion of affected women to break the cycle of depression and HIV infection interaction. It is documented that integrating mental health services into primary care may be the most viable way of closing treatment gap for mental health in low resource settings [23] in order to achieve the United Nations Sustainable Development Goals (SDGs). An important step in this direction is the incorporation of the capacity to prevent, recognise and treat depression within antenatal care [27]. This may help to meet the immediate mental health needs of a pregnant woman, ensure better maternal and child outcomes and contribute towards the success of HIV/AIDS services [24].

Integrated antenatal services aimed at identifying and treating women with antenatal depression are needed because antenatal care is typically the first and only time of interaction with the health care system for many women in low resource settings [23]. As such, antenatal care visits provide critically important opportunities for mental health interventions to occur. But antenatal depression is often underdiagnosed by midwives because there are no depression screening instruments at antenatal clinics in Malawi [15]. Nonetheless, a previous study proposed SPADe, a locally developed protocol as suitable for screening depression among pregnant women in antenatal clinics in Blantyre District [17]. It is documented that locally developed protocols may be more acceptable to clinicians and consequently more likely to be adopted and used in practice [28]. However, utilisation of SPADe in clinical practice to implement screening of depression in antenatal clinics has never been tested before.

Therefore, the primary aim of this study is to assess the feasibility of screening for depression by midwives using SPADe in antenatal clinics in Blantyre District. The secondary aim was to assess acceptability and fidelity of screening for depression by midwives using SPADe in antenatal clinics in Blantyre District. In this study, acceptability refers to what extent is a new idea, programme, process or measure judged as suitable, satisfying or attractive to programme deliverers or programme recipients [29] while feasibility means determining whether an intervention can be shaped to be relevant and sustainable by identifying what aspects need modification and how changes might occur [29]. Furthermore, fidelity of treatment refers to the confirmation that the manipulation of the independent variable occurred as planned to ensure that fair, powerful and valid comparisons of replicable treatments can be made [30].

### Conceptual model

**Fig. 1:**
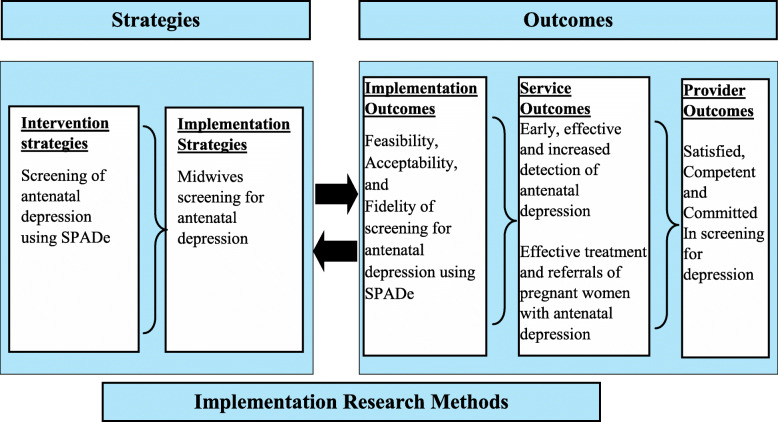
Conceptual model of implementation research. Adapted from Proctor et al. (2009) [31]

This study will draw on a Conceptual Model of Implementation Research (CMIR) by Proctor and colleagues [31] as its underpinning (Fig. 1). The major concepts of CMIR are implementation strategies and outcomes which are linked. The model proposes evidence-based intervention strategies and separate strategies for implementing those interventions in usual care. Furthermore, the CMIR proposes three distinct but interrelated types of outcomes namely: implementation, service and client outcomes. It also allows for involvement of multiple stakeholders at multiple levels of implementation processes. The CMIR recommends that a relationship between intervention strategies and implementation strategies should be empirically tested because various intervention strategies encounter unique implementation challenges.

### Application of conceptual model to the study

Antenatal depression is often underdiagnosed by midwives because there are no depression screening instruments at antenatal clinics in Blantyre District in Malawi [15]. In this study, an implementation strategy for screening antenatal depression (SPADe) by midwives will be tested in three antenatal clinics in Blantyre District [17]. This study will use clinician implementation team strategy by Proctor and colleagues [32] that will utilise a team of midwives (actor) to implement SPADe, a clinical innovation. It will involve (action) reflecting on the implementation effort, sharing of lessons learned, support learning and proposition of changes to be implemented. The targets of the action will be midwives that will be newly trained in screening for antenatal depression using SPADe. Midwives will be equipped with knowledge about how to use the SPADe to screen for depression in antenatal clinics and collaborate effectively with mental health specialists so that pregnant women are able to access mental health care within antenatal clinics. The research team will meet the midwives for the first time to facilitate the commencement of screening for depression in antenatal clinics within 2 weeks of initial training (temporality). Then, the research team will go to antenatal clinics once fortnightly for 4 h for the 4 months (dose) to conduct supportive supervision to the midwives. This will be done to facilitate uptake of screening using SPADe among pregnant women, ensure that midwives are implementing SPADe faithfully and ensure its sustainability (implementation outcome). The proposed clinician implementation team strategy is relevant for this study because it will enhance cooperative learning among midwives and the researchers [33].

The implementation outcomes for this study will encompass feasibility, acceptability and fidelity of utilising SPADe in screening for antenatal depression. The service outcomes for this study will be twofold: (1) early, effective and increased detection of antenatal depression and (2) effective treatment and referrals of pregnant women with antenatal depression. The service provider’s outcome that will be measured in this study will be midwives’ satisfaction, competence and commitment in screening of antenatal depression.

## Methods

This study will be an implementation research which will consist of scientific investigations that will support the movement of evidence-based, effective health care approach, SPADe, from the clinical knowledge base into routine use [31]. This study will employ multi-methods to assess the feasibility, acceptability and fidelity of screening for antenatal depression using SPADe in three phases: phase 1 will introduce SPADe in antenatal clinics in Blantyre District where screening of depression almost none existent; phase 2 will implement screening of depression using SPADe in antenatal clinics in Blantyre District; and phase 3 will evaluate the screening of antenatal depression using SPADe to establish its feasibility, acceptability and fidelity in antenatal clinics in Blantyre District.

### Phase 1: Introducing of screening for antenatal depression using SPADe

#### Setting for the study

The study setting will be Blantyre District in Malawi and includes the antenatal clinics of the following health centres: Chileka, Mpemba and Ndirande. These are government health facilities which provide free health care services to the majority of people in rural and urban areas of Blantyre District. Midwives run antenatal clinics at these health facilities. The clinics are conducted during morning hours from Monday to Friday every week. These settings are suitable for this study because they have established antenatal clinics where pregnant women can be easily accessed. The three antenatal clinics are included in this study because they previously contributed data that informed development of SPADe [17]. The antenatal attendance of pregnant women at these health facilities for the period June 2018 to July 2019 ranged from 1292 to 11,498.

#### Study population

The target population will include all midwives working in antenatal clinics at Chileka (21), Mpemba (15) and Ndirande Health Centres (26) making a total of 62 midwives. All 62 midwives will participate in this study.

#### Process of introducing screening of antenatal depression using SPADe

The study will be conducted from July 2020 to March 2020. Prior to introduction of screening for antenatal depression using SPADe in antenatal clinics, the PI will hold four meetings with Leadership at District Health Office level (1) and Health Centre Level (3) in July 2020. The meetings will aim at orienting the leaders about the study and also seek their buy-in. In this study, the screening of antenatal depression by midwives using SPADe will be introduced in the three aforementioned health centres. The researcher will explain the study to all midwives and invite them to participate in this study before the commencement of training. Those who will accept to participate will give a written consent. The midwives will implement screening of antenatal depression and referrals after receiving training on the use of SPADe [17]

In August 2020, the PI, an Advanced Practice Mental Health Nurse, with the help of collaborators will train all midwives (62) in the three health centres in screening of antenatal depression using SPADe. In addition, 4 mental health nurses from Queen Elizabeth Central Hospital will also participate in this training resulting in a total of 66 participants. Two training sessions for the trainer of trainers will be conducted within Blantyre for 3 days and will involve one day for theory and 2 days for practice at one antenatal clinic to create a critical mass. Each training will include half of the midwives from each health centre. This would allow midwives to participate in the training without disrupting the delivery of services in these facilities.

Prior to the training, all participants will be asked to answer a pre-test questionnaire (baseline) on their commitment and competency in screening for antenatal depression including the support they receive (Additional file 1), and it will be repeated at the end of the training (post-test). These trained midwives will be expected to train on the job new staff about screening of antenatal depression using SPADe training materials as a way of realising a multiplier effect. During the practical sessions of training, midwives will be supervised by the PI and collaborators.

All midwives (62) will be required to use SPADe to screen for antenatal depression and determine a need for treatment or referral among pregnant women. The SPADe is specifically designed for pregnant women with depression, and it is not intended to cover the whole spectrum of pregnant women with other mental disorders. This screening protocol focuses on improving the quality and accessibility of maternal mental health care by integrating routine screening for depression into antenatal services so that pregnant women with, or at risk of, depression are timeously detected and the appropriate treatment can commence. The SPADe is intended to reflect optimum practice in routine screening for depression and the management of pregnant women at risk or with depression in antenatal clinics in Malawi. The components of the SPADe include antenatal services and antenatal care assessment, midwives’ functions, screening instruments and mental health assessment. It also includes an algorithm which outlines steps for screening and treatment pathways (Additional file 2). After completing all training sessions in August 2020, all the trained midwives from the three health centres will be equipped with materials to start screening for depression using SPADe. They will also be charged with the responsibility of teaching new staff in the antenatal clinics on how they can effectively screen in their settings.

### Phase 2: Implementation of screening for depression using SPADe in antenatal clinics

In September 2020, the three health centres will prepare to implement screening of antenatal depression using SPADe. Meetings will be held with leadership at the facilities, midwives and mental health nurses to integrate the screening of depression in their local antenatal clinics. From September to December 2020, screening of antenatal depression using SPADe will be launched in the three health centres. Pregnant women at any stage of gestation will be screened for depression at the initial booking at the antenatal clinic.

Midwives will initially administer the 3-Item Screener [15, 17] to pregnant women during history taking (Additional file 3) and those who will screen positive on the 3-Item Screener will be interviewed further using Self Reporting Questionnaire (SRQ) 20 (Additional file 3) immediately [15, 17]. Pregnant women who will screen positive on SRQ 20 will be further interviewed by the PI or a mental health nurse to confirm the presence of major depressive episode on the same day using the major depression module of the Mini International Neuropsychiatric Interview (MINI) [34]. The PI and visiting mental health nurses will also provide therapy (antidepressants and psychosocial counselling) to all pregnant women confirmed as having depression in accordance with SPADe and Malawi Standard Treatment Guidelines [35].

During the entire implementation period of screening for depression, midwives will be receiving support in terms of continuous supervision from the PI, collaborators and 4 visiting mental health nurses from Queen Elizabeth Central Hospital Psychiatric Unit. A focal person for screening of antenatal depression using SPADe (midwife in charge of the antenatal clinic) in each clinic will be providing feedback on screening rates at staff meetings and via posters and relevant Health Management Information System (HMIS) registers.

### Measuring feasibility, acceptability and fidelity of screening for depression among midwives

This will be both a quantitative and qualitative survey that will be conducted in antenatal clinics at Chileka, Mpemba and Ndirande Health Centres. The study sample will include all 62 midwives who would have received training in screening using SPADe and will be participating in screening for antenatal depression (Chileka = 21, Mpemba = 15 and Ndirande = 26) will be surveyed three times between September and December 2020. The surveys will focus on measuring feasibility, acceptability and fidelity of screening for depression using SPADe.

The PI with the help of two research assistants (Registered Midwives) will collect data from midwives. Prior to data collection, the research assistants will receive two days training that will include an overview of the research study, review of data collection techniques and instruments, practice on the use of data collection instruments and a discussion on ethical issues pertaining to the study. The PI and a research assistant will hand a self-administered semi-structured questionnaire to each respondent and ask them to answer it immediately. The PI and research assistant will collect all completed questionnaires immediately after they have been completed.

#### Measuring feasibility of using SPADe by midwives

The adapted Structured Assessment of FEasibility (SAFE) Version 1.1 [36] will be used to assess the extent to which SPADe is feasible for implementation in antenatal clinics. Midwives will be asked to indicate their level of agreement with each of the 16 statements by choosing one of the following possible responses: yes, partial, no or unable to rate (Additional file 4). Exactly one rating is made for each item. It is recommended that no overall summary score is used, as barriers and facilitators differ in their importance depending on the context.

A prospective questionnaire to assess feasibility of the SPADe among 62 midwives at the three selected health centres in Blantyre District will be administered to participants. This study will use census (62 midwives) because all midwives rotate in antenatal clinics in all health centres. They will anonymously complete the instrument (SAFE) at three evaluation moments during SPADe implementation period, September 2020 to December 2020. The implementation process of SPADe will be continuously updated by posters and supervision. We will assess feasibility of the SPADe at the beginning of the implementation of screening in September 2020 and the second moment will be halfway the implementation (end October 2020) and the last moment will be at the end of the implementation of screening (December 2020). As feasibility outcomes, we expect that (1) ≥80% of participants will be participating in screening for antenatal depression and (2) increase in the number of pregnant women detected with depression in antenatal clinics by December 2020. The following criteria will be used to determine the success of feasibility: (1) ongoing supportive supervision by mental health nurses will be provided to midwives in antenatal clinics regularly during the implementation of SPADe and (2) the full SPADe manual will be available from the corresponding author. We will compute descriptive statistics for barriers and enablers for the implementation of SPADe at three points of data collection. Cross tabulations will be used to compare barriers and enablers for the implementation of SPADe that will be identified at three points of data collection. To evaluate feasibility of using SPADe for screening antenatal depression, the primary outcome measures will be (1) percentage of midwives (≥ 80%) who will participate in screening by December 2020 and (2) increase in percentage (from 0%) of pregnant women detected with depression by midwives. All measures will be assessed at each time point. The individual's midwife 3 ratings of the feasibility questionnaire will be analysed separately from the collective set of data that will be collected for the general attitude of midwives assess their change in attitude while gaining more confidence in implementing the screening protocol between September and December.

#### Measuring acceptability of using SPADe by midwives

The Ottawa Acceptability of Decision Rules Instrument (OADRI) that was designed to measure the acceptability of clinical decision rules will be used to assess acceptability of SPADe [37]. Midwives will be asked to indicate their level of agreement with each of the 12 statements on a 6-point scale ranging from 1 (strongly disagree) to 6 (strongly agree), or indicating ‘no opinion/don’t know’ (Additional file 4). The first 7 items have been phrased such that a higher number indicated greater acceptability while the last 5 have been phrased so that a higher number indicated less acceptability. The final total score of the instrument will consist of the average of all 12 items (ranging from 0 to 6). Non-completed items will be excluded from the final total scores. The average of the remaining items will serve as the instrument’s score. Midwives who will complete less than 8 of the 12 items will be considered as not having completed the instrument and will be excluded from the final analyses. Items for which ‘no opinion/don’t know’ was selected will be coded as the middle of the scale [37].

A prospective questionnaire evaluation among 62 midwives at the three selected health centres in Blantyre District will be conducted. They will anonymously complete the instrument (OADRI) at three evaluation moments during SPADe implementation period, September 2020 to December 2020. The implementation process of SPADe will be continuously updated by posters and supervision. The first evaluation moment will take place at the beginning of the implementation of screening in September 2020 and the second moment will be halfway the implementation (end October 2020) and the last moment will be at the end of the implementation of screening (December 2020).

We will calculate average item scores and greater acceptability will be indicated by higher scores. To test differences between the item scores of the OADRI during the three moments of the screening implementation, we will use linear regression analyses. We will test changes in average item scores within the suggested original categories of OADRI items [37]: (1) aspects of innovation, items 1–4; (2) decision-maker, items 6–9 and 11; and (3) environment, items 5, 10, and 12. The individual’s midwife 3 ratings of the acceptability questionnaire will be analysed separately from the collective set of data that will be collected for the general attitude of midwives assess their change in attitude while gaining more confidence in implementing the screening protocol between September and December.

#### Measuring fidelity of using SPADe by midwives

At the start of the project, all midwives (62) will be trained in the implementation of SPADe in a three days training that will include a practical component. They will receive biweekly supervision from a mental health specialist at antenatal clinics that will be implementing SPADe. A refresher training for all midwives (62) will take place at the midpoint (end October 2020) of implementing SPADe.

A prospective evaluation of fidelity in using SPADe among 62 midwives will be conducted using a questionnaire that has been adapted from Bellg and colleagues [38] recommendations for enhancing treatment fidelity in behaviour change studies (Additional file 5). These recommendations include study design, training providers, delivery of treatment, receipt of treatment and enactment of treatment skills [38]. This study will focus on two recommendations namely: training providers and delivery of treatment because the other three are beyond the scope of this study. Training of providers is an important area of fidelity that will be assessed to ensure that midwives have been satisfactorily trained to implement SPADe with pregnant women. Literature suggests that the adequacy of training to implement an intervention should be evaluated and monitored on an individual basis both during and after the training process [38]. Delivery of treatment will focus on treatment fidelity processes that will monitor and improve implementation of SPADe so that it is delivered as intended. It is documented that even well-trained interventionists may not always deliver an intervention protocol effectively when clinical circumstances or their training or involvement in other types of interventions interfere with their doing so [38]. The goal will be to check for midwives’ adherence to implementation of SPADe in clinical practice as intended.

Midwives will anonymously complete the questionnaire at the beginning of the implementation of screening in September 2020 and the second moment will be halfway the implementation (end October 2020) and the last moment will be at the end of the implementation of screening (December 2020). Furthermore, the researchers will do the following to assess fidelity:

- Fidelity strategies for monitoring and improving midwives training

The researchers will keep field notes about the training that midwives will receive prior to the implementation of SPADe. The field notes will check and confirm if the training will be conducted as described in this section:

i. The study will use a standardized training to equip all midwives with knowledge and skills for using SPADe in screening antenatal depression.
ii. All the 62 midwives will receive the same training in screening using SPADe together.
iii. A standardised SPADe training manual will be used during training.
iv. SPADe training will accommodate midwives of different cadres regardless of their different levels of experience.
v. Midwives will be involved in structured practice and role-playing during SPADe training
vi. Standardised pregnant women will be used during SPADe training.
vii. These midwives will have an opportunity to observe SPADe implementation with standardised pregnant women.
viii. Same instructors will be used to train all midwives
ix. SPADe training will be videotaped in case there would be a need for future training for other midwives.

The study will ensure midwives’ skill acquisition by assessing midwives’ adherence to screening using SPADe by:

i. Using a skills acquisition checklist (Additional file 6).
ii. Midwives writing an exam pre- and post-SPADe training (Additional file 1).

The researchers will minimise ‘drift’ in midwives’ skills will by:

i. Conducting regular booster sessions about SPADe with midwives
ii. Conducting weekly supervisions and periodic meetings on screening using SPADe
iii. Allowing easy access to project staff to ask questions about screening using SPADe
iv. Keeping field notes about booster sessions, supervisions, meetings and availability of project staff to midwives
v. Having midwives complete self-report questionnaires to evaluate the use of SPADe at the end of the study (Additional file 5).

b. Fidelity strategies for monitoring and improving the implementation of SPADe

Researchers will control provider differences by:

i. Ensuring that midwives are screening all pregnant women using SPADe without being selective and field notes will be recorded.
ii. Conducting a qualitative and quantitative survey at the end of the study (Additional file 5).

Researchers will reduce differences within the screening process by:

i. Providing a scripted SPADe to all midwives that will be used by during screening of pregnant women
ii. Audio or video recording all encounters with midwives to ensure adherence to SPADe
iii. Checking errors of omission and commission in the implementation of SPADe which will be recorded as field notes.

We will compute descriptive quantitative data and content analysis will be used to analyse qualitative data about fidelity in implementation of SPADe in antenatal clinics. The researchers will rate audio/video clips on their encounter with midwives or screening sessions conducted by midwives using a checklist (Additional file 6)

### Phase 3: Evaluation of screening for antenatal depression using SPADe

In December 2020, 4 months after implementing screening of antenatal depression using SPADe, a multi-methods evaluation (quantitative and qualitative survey) of the programme will be conducted. Midwives will be asked about their views on screening of antenatal depression using SPADe and how they accomplished this task, and screening rates and how many women were identified as possibly depressed will be determined. The evaluation will focus on feasibility, acceptability and fidelity of screening for antenatal depression using SPADe among midwives.

This will be a quantitative and qualitative survey. Quantitative data will be used to evaluate implementation outcomes of screening antenatal depression and qualitative data to evaluate the process of implementing depression screening using SPADe [39]. Furthermore, qualitative data will be used to provide a depth of understanding about screening of antenatal depression and quantitative data will provide a breadth of understanding about screening for antenatal depression. This study will use all 62 midwives who would have received training in screening using SPADe and would have been working in antenatal clinics during the study period. The instrument that will be used to collect data in this study was developed by the PI by adopting and adapting questions that were previously used by other researchers to assess acceptability, feasibility and fidelity of screening depression [40] or other conditions among health professionals [41] (Additional file 5). The instrument will be a self-administered semi-structured questionnaire that will be used to assess how midwives viewed the implementation of depression screening in antenatal clinics. The instrument will include both closed questions (1–7, 10–11) and directive free-text questions (8–9, 12–19) (Additional file 5). Directive free-text questions will be an appropriate method for gathering the opinions of contextual issues that are considered pertinent by respondents of a survey [42, 43]. These questions will enable midwives to express their opinions and provide useful contextual information at the time the survey will be administered [44]. The instrument will be in English because all midwives are expected to be conversant with English by virtue of their training.

The PI with the help of a research assistant will conduct two data collection sessions within Blantyre District in December 2020. A total of 31 midwives (Chileka = 11, Mpemba = 7 and Ndirande = 13) will be invited to the first data collection session. Then, 2 days later, a second group of 31 midwives (Chileka = 10, Mpemba = 8 and Ndirande = 13) will also be called to a data collection session within Blantyre District as well. These midwives who will participate in screening for depression will respond to a survey at the end of 4 months period. The PI and a research assistant will hand a self-administered semi-structured questionnaire to each respondent and ask them to answer it right there without consulting others. The PI and research assistant will collect all completed questionnaires before respondents leave the meeting room. Finally, the PI will thank all respondents for taking part in the study.

This study will adopt recommendations for reporting free-text comments that will involve reporting the minimum and maximum length of sentences made by participants, the number of participants who will make comments within each theme and the number of participants who will fail to make any free-text responses [45, 46]. Content analysis will be used to analyse free-text comments [47, 48]. The comments will be read multiple times in order to gain immersion in the data. Content-characteristic words in free-text comments will be identified and free-text comments sorted into categories, from which emergent themes will be identified. Exemplar comments with the descriptive text accompanying each theme will be used in content analysis [47]. The data analysis will also include a quantitative summary of the themes encompassing the midwives’ responses.

Quantitative data will be analysed using Statistical Package for Social Sciences (SPSS) 25 with the help of a statistician. Descriptive statistics including means, standard deviations, percentages and frequencies will be used to summarise data. Potential differences in demographic characteristics will be examined using Pearson’s chi-squared test and statistical significance level will be set at *P* < 0.05 with 95% confidence interval. Binary logistic regression models will be used to generate factors associated with feasibility and acceptability of screening for depression using SPADe.

### Ethical considerations

The study proposal received ethics approval from the College of Medicine Research and Ethics Committee (COMREC) at the University of Malawi (P.02/20/2935). The PI and research assistant will explain the nature and benefits of the study to pregnant women and midwives before they are recruited to participate in the study. They will ensure that they minimise harm to pregnant women and midwives who will participate in this study. Participants’ names will not form part of demographic data that will be collected, thus respecting their privacy and maintaining confidentiality. Midwives will be informed that they will be offered Malawi Kwacha equivalent of $10.00 (K7 500.00) for participating in the study. Furthermore, they will be informed that the information which they will provide will be utilised in making recommendations about maternal mental health care targeting pregnant women. Participants will be informed that only aggregated data will be analysed and disseminated. They will also be informed that all hard copies of data collected will be locked in a cabinet of the PI at Kamuzu College of Nursing and will be incinerated after 5 years. As for electronic data, it will be secured by a password known only to the researcher and would be deleted from the storage device after 5 years.

Midwives will be informed that their participation in the study is voluntary and those who accept to participate will be asked to give a written informed consent. They will be told that they will be free to withdraw at any time if they felt uncomfortable about any aspect during the course of the study. As such, evaluation of screening for antenatal depression using SPADe will only include data collected from midwives who will participate in the actual implementation of SPADe in antenatal clinics. They will also be told that refusing to join the study will not have any effect on their job because the PI is not an employee of the Ministry of Health.

## Discussions

This study will establish and document feasibility, acceptability and fidelity of screening for depression by midwives using SPADe in antenatal clinics in Blantyre District. It is expected that midwives will develop more confidence in detecting and dealing with antenatal depression. Consequently, there will be increased numbers of pregnant women detected with depression by midwives and increased accessibility to mental health care by pregnant women in antenatal clinics. A limitation of this study is that it will be conducted in Blantyre District only which may not be representative of Malawi cultures. However, Blantyre has both rural and urban settings such that it has features of rural and urban districts in Malawi. Midwives may feel overwhelmed with the task of screening for depression. However, this challenge will be lessened by using mental health nurses to provide treatment to pregnant women with depression. Midwives will also be assisted through continuous supportive supervision throughout the study period. The study design is also limited because screening of women at the initial booking only will not allow pregnant women who will screen ‘negative’ for depression to have another chance to screen ‘positive’ in subsequent visits if they were to develop depression later. Furthermore, it would also be hard for midwives to re-screen ‘negative’ cases in subsequent visits as this will be a new task for them. This will probably affect midwives’ feasibility scores and validity of the study because midwives may be required to re-screen ‘negative’ patients in subsequent visits in real life.

## Supplementary Information

**Additional file 1.** Pretest/posttest for SPADe training received by midwives.

**Additional file 2.** Algorithm for screening Antenatal depression.

**Additional file 3.** Screening instruments for antenatal depression.

**Additional file 4.** Questionnaire.

**Additional file 5.** Evaluation of screening for antenatal depression using SPADe among midwives.

**Additional file 6.** Skills acquisition checklist on implementation of SPADe.

## Data Availability

The data collection instruments that will be used in this study are available as additional files or may be requested from the corresponding author. Anonymised versions of the datasets that will be used and analysed in the proposed study will be available from the corresponding author on reasonable request

## Abbreviations

CMIR: Conceptual Model of Implementation Research
COMREC: College of Medicine Research and Ethics Committee
HMIS: Health Management Information System
OADRI: Ottawa Acceptability of Decision Rules Instrument
SAFE: Structured Assessment of Feasibility
SDGs: Sustainable Development Goals
SPADe: Screening Protocol for Antenatal Depression
SRQ 20: Self Reporting Questionnaire 20
