# Supplementary material for: Protocol for assessing feasibility, acceptability and fidelity of screening for antenatal depression (FAFSAD) by midwives in Blantyre District, Malawi
Source: Pilot Feasibility Stud. 2021 Jan 26;7:32. doi: 10.1186/s40814-021-00775-6 (PMC7836563; doi:10.1186/s40814-021-00775-6)
Supplement: Supplementary file 1 — Additional file 1. Pretest/posttest for SPADe training received by midwives. [file 40814_2021_775_MOESM1_ESM.docx]

**Attachment 1: Pretest/posttest for SPADe training received by midwives**

**INITIALS: __________________ FACILITY**: ___________________ **CODE**________

**SECTION A**

**Demographic Characteristics**

1. What is your age in years? _______________
2. How many years you have been practicing as a midwife? _______________

*Please circle the option which applies to you*

1. Gender: 1. Male

2. Female

1. Which nursing/midwifery cadre do you belong to? (circle all tha apply)

1. Registered Nurse/Midwife

2. Nurse Midwife Technicians

3. Enrolled Nurse Midwife

4. Enrolled Psychiatric Nurse

5. Other specify______________

1. Did you receive any form of maternal mental health lectures as part of your training? Yes No

**SECTION B**

**Instruction**: Indicate whether the following statements are true or false by ticking in the appropriate box corresponding to each statement

| **SN** | **Statement** | **True** | **False** |
| --- | --- | --- | --- |
|  | The aim of the SPADe is to improve health of pregnant women and child they are expecting |  |  |
|  | SPADe ensures standardised approach for detecting and dealing with pregnant women who have depression |  |  |
|  | SPADe helps midwives to detect pregnant women with depression early |  |  |
|  | SPADe will improve collaboration between antenatal services and mental health services |  |  |
|  | Midwife should administer 3 Item screener to all women initially at booking visit |  |  |
|  | Midwife can use 3 Item screener to make a diagnosis of depression in antenatal clinics |  |  |
|  | Optimum cut off score for 3 Item screener is 2 |  |  |
|  | All pregnant women who do not have depression score less than 2 on the 3 Item screener |  |  |
|  | Midwife should not bother about pregnant women who score 1 or 0 on the 3 Item screener |  |  |
|  | Midwife should only administer to SRQ 20 to pregnant women who score 2 or 3 on the 3 Item screener |  |  |
|  | Midwife should refer for mental health assessment any pregnant woman who scores 10 or more on SRQ 20 |  |  |
|  | Maximum score a pregnant woman can achieve on the SRQ 20 is 20 |  |  |
|  | A midwife should add up all questions that a pregnant woman has anwered ‘Yes’ on the SRQ 20 to calculate a score achieved by a pregnant woman |  |  |
|  | It is necessary that a midwife asks a pregnant woman all questions on the 3 Item screener or SRQ 20 |  |  |
|  | It is not a responsibility of a midwife to follow up and monitor treatment of all pregnant women diagnosed with depression |  |  |
|  | Midwife should engage in watchful waiting and targeted psychoeducation of all pregnant women who score ≤1 on the 3 Item screener or ≤9 on SRQ 20 |  |  |
|  | Pregnant women diagnosed with depression should be commenced on treatment by mental health specialists |  |  |
|  | Midwives should not receive supportive supervision from mental health specialists |  |  |
|  | Pregnant woman who scores 1 specifically on questions 16 or 17 of SRQ 20 should be immediately refered to a mental health specialist |  |  |

**THANK YOU VERY MUCH FOR YOUR PARTICIPATION**
