# Supplementary figures and images for: Protocol for assessing feasibility, acceptability and fidelity of screening for antenatal depression (FAFSAD) by midwives in Blantyre District, Malawi
Source: Pilot Feasibility Stud. 2021 Jan 26;7:32. doi: 10.1186/s40814-021-00775-6 (PMC7836563; doi:10.1186/s40814-021-00775-6)

**Attachment 2: Algorithm for screening Antenatal depression**


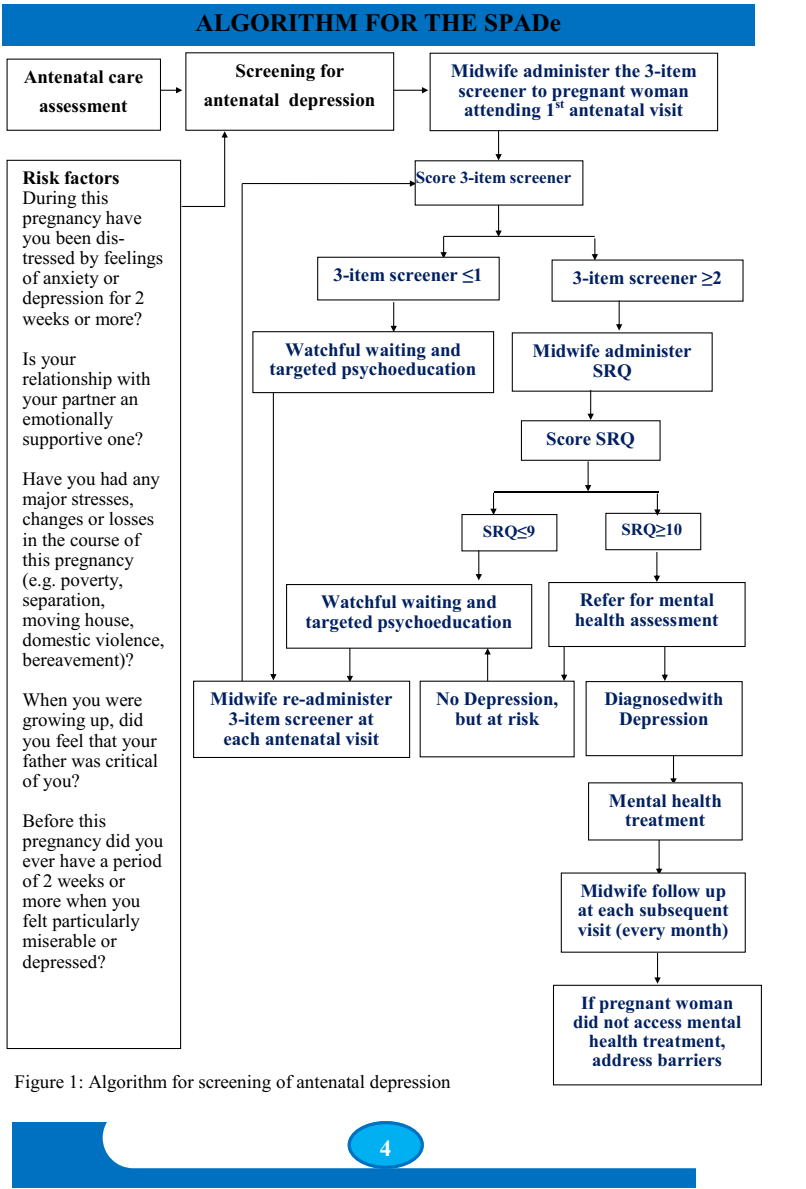

Supplement: Supplementary file 2 — Additional file 2. Algorithm for screening Antenatal depression. [file 40814_2021_775_MOESM2_ESM.docx]
