# Supplementary material for: Protocol for assessing feasibility, acceptability and fidelity of screening for antenatal depression (FAFSAD) by midwives in Blantyre District, Malawi
Source: Pilot Feasibility Stud. 2021 Jan 26;7:32. doi: 10.1186/s40814-021-00775-6 (PMC7836563; doi:10.1186/s40814-021-00775-6)
Supplement: Supplementary file 3 — Additional file 3. Screening instruments for antenatal depression. [file 40814_2021_775_MOESM3_ESM.docx]

**Attachment 3: Screening instruments for antenatal depression**

**Date_______________ Setting____________________** **CODE**________

**Age______________Gestation ____________ Parity___________HIV status_______**

**Number of Children___________ Occupation___________________**

**Level of education____________________Marital Status_________________**

**PART A: Administer to all women at booking antenatal visit**

**The 3-item screener** **for assessing depression in pregnant women**

Now I will ask you quick questions which detects pregnant women with depression. Answer “Yes” or “No” on each and every question I will ask you. Please ask if you have not understood a question and I will explain to you what that question means.

Tick yes/no, as applicable against the following questions

| **S.N** | **Question** | **Yes** | **No** |
| --- | --- | --- | --- |
| 1 | During the past month, have you been bothered by feeling down, depressed or hopeless? |  |  |
| 2 | During the past month, have you been bothered by little interest or pleasure in doing things? |  |  |
| 3 | Are you depressed? |  |  |

**PART B: Administer only to women who answer ‘YES’ to two or more questions in Part A imediately**

**SRQ 20**: Now I will ask you about how you have been feeling and thinking in the past four weeks. Answere “Yes” or “No” on each question that I will ask you. If you are not sure, answer in a way that is closer to how you have been feeling. If you do not understand the question, please ask and I will explain to you what that question means.

Tick “Yes” or “No”, as applicable against the following questions

| **S.N** | **Question** | **Yes** | **No** |
| --- | --- | --- | --- |
| 1 | In the past four weeks, did you often have headaches? |  |  |
| 2 | In the past four weeks, was your appetite poor? |  |  |
| 3 | In the past four weeks, did you sleep badly? |  |  |
| 4 | In the past four weeks, were you easily frightened? |  |  |
| 5 | In the past four weeks, did your hands shakes? |  |  |
| 6 | In the past four weeks, did you feel nervous, tense or worried? |  |  |
| 7 | In the past four weeks, was your digestion poor? |  |  |
| 8 | In the past four weeks, did you have trouble thinking clearly? |  |  |
| 9 | In the past four weeks, did you feel unhappy? |  |  |
| 10 | In the past four weeks, did you cry more than usual? |  |  |
| 11 | In the past four weeks, did you find it difficult to enjoy your daily activities? |  |  |
| 12 | In the past four weeks, did you find it difficult to make decisions? |  |  |
| 13 | In the past four weeks, was your daily work suffering? |  |  |
| 14 | In the past four weeks, were you unable to play a useful part in life? |  |  |
| 15 | In the past four weeks, have you lost interest in things? |  |  |
| 16 | In the past four weeks, did you feel that you are a worthless person? |  |  |
| 17 | In the past four weeks, has the thought of ending your life been on your mind? |  |  |
| 18 | In the past four weeks, did you feel tired all the time? |  |  |
| 19 | In the past four weeks, did you have uncomfortable feelings in your stomach? |  |  |
| 20 | In the past four weeks, were you easily tired? |  |  |

**Send all women who have answered ‘YES’ to 10 or more question in Part B or have anwered ‘YES’ to question 16 or 17 for further diagnostic assessment by a mental health nurse within Antenatal Clinic**

**Screening instruments for antenatal depression (Chichewa version)**

**Date_______________ Setting____________________** **CODE**________

**Age______________Gestation ____________ Parity___________HIV status_______**

**Number of Children___________ Occupation___________________**

**Level of education____________________Marital Status_________________**

**GAWO A: Funsani mafunsowa kwa amayi onse pamene abwera kudzayamba sikelo**

**The 3-item screener** **yoyezera amayi apathupi omwe ali ndi matenda okhumudwa**

Tsopano ndikufunsani mafunso achidule amene amadziwitsa ngati amayi apathupi ali ndi nthenda yokhumudwa. Mundiyankhe kuti “eya” kapena “ayi” pa funso lirilonse lomwe ndi kufunseni. Ngati funso simunalimvetse bwino, chonde funsani ndipo ndidzakufotokozerani chomwe funsolo likutanthauza.

*Chongani yankho loyenera*

|  | **Funso** | **Eya** | **Ayi** |
| --- | --- | --- | --- |
| 1 | Kodi mmwezi wapitawu mwakhala mukuvutika mumtima mwanu chifukwa chaku khumudwa kapena kukhala opanda chiyembekezo? |  |  |
| 2 | Kodi mmwezi wapitawu mwakhala mukuvutika mumtima mwanu chifukwa chokhala opanda chidwi kapena kusasangalatsidwa pochita zinthu? |  |  |
| 3 | Kodi ndinu okhumudwa? |  |  |

**GAWO B: Funsani mafunsowa kwa amayi okhawo amene ayankha kuti ‘EYA’ mafunso awiri kapena kuposera apo mu Gawo A**

**SRQ 20**: Tsopano ndikufunsani za momwe mwakhala mukumamvera mumtima mwanu ndi maganizo omwe mwakhala muli nawo mmasabata anayi omwe apitawa. Mundiyankhe kuti “Eya” kapena “Ayi” pafunso lirilonse lomwe ndi kufunseni. Ngati mukukaikira, yankhani mofanizira ndi momwe mwakhala mukumvera. Ngati funso simunalimvetse bwino, chonde funsani ndipo ndidzakufotokozerani chomwe funsolo likutanthauza.

*Chongani yankho loyenera*

| **SN** | **Funso** | **Eya** | **Ayi** |
| --- | --- | --- | --- |
| 1 | M'masabata anayi apitawa, kodi mumamva kupweteka mutu pafupipafupi? |  |  |
| 2 | M'masabata anayi apitawa, kodi simumakhala ndi chilakolako cha chakudya? |  |  |
| 3 | M'masabata anayi apitawa, kodi mumavutika kugona usiku? |  |  |
| 4 | M'masabata anayi apitawa, kodi simumachedwa kututumutsidwa? |  |  |
| 5 | M'masabata anayi apitawa, kodi manja anu amanjenjemera? |  |  |
| 6 | M'masabata anayi apitawa, kodi mumakhala ndi nkhawa, mantha kapena madandaulo? |  |  |
| 7 | M'masabata anayi apitawa, kodi mumadzimbidwadzimbidwa? |  |  |
| 8 | M'masabata anayi apitawa, kodi mumakhala ndi vuto kuganiza bwinobwino? |  |  |
| 9 | M'masabata anayi apitawa, kodi mumakhala osasangalala kapena osakondwa? |  |  |
| 10 | M'masabata anayi apitawa, kodi mumaliralira pafupipafupi ndipo koposera muyeso? |  |  |
| 11 | M'masabata anayi apitawa, kodi mumaona ngati ndi chinthu chokuvutani kusangalatsidwa ndi zinthu zimene mumapanga tsiku ndi tsiku? |  |  |
| 12 | M'masabata anayi apitawa, kodi mumakhala ndi vuto kupanga maganizo kapena kumanga mfundo? |  |  |
| 13 | M'masabata anayi apitawa, kodi ntchito zanu za tsiku ndi tsiku sizimayenda bwino? |  |  |
| 14 | M'masabata anayi apitawa, kodi mumalephera kupanga zinthu za phindu kapena zofunikira m’moyo wanu? |  |  |
| 15 | M'masabata anayi apitawa, kodi munasiya kukhala ndi chidwi mu zinthu zosiyanasiyana? |  |  |
| 16 | M'masabata anayi apitawa, kodi mumaziona ngati ndinu munthu wopanda ntchito kapena wosafunikira? |  |  |
| 17 | M'masabata anayi apitawa, kodi maganizo odzipha anayamba akubwereranipo? |  |  |
| 18 | M'masabata anayi apitawa, kodi mumamva kapena kukhala otopatopa nthawi zonse? |  |  |
| 19 | M'masabata anayi apitawa, kodi mumakhala ndi vuto losamva bwino m’mimba? |  |  |
| 20 | M'masabata anayi apitawa, kodi simumachedwa kutopa? |  |  |

**Atumizeni amayi onse amene ayankha kuti ‘EYA’ mafunso 10 kapena kuposera apo mu Gawo B komanso ngati ayanka ‘EYA’ funso 16 nsi 17 kwa namwino owona za nthanzi la mmalingaliro kuti akawayeze mozama ku malo a sikelo omwewo**
