# Supplementary material for: Protocol for assessing feasibility, acceptability and fidelity of screening for antenatal depression (FAFSAD) by midwives in Blantyre District, Malawi
Source: Pilot Feasibility Stud. 2021 Jan 26;7:32. doi: 10.1186/s40814-021-00775-6 (PMC7836563; doi:10.1186/s40814-021-00775-6)
Supplement: Supplementary file 4 — Additional file 4. Questionnaire. [file 40814_2021_775_MOESM4_ESM.docx]

**Attachment 4: Questionnaire**

**CODE/INITIALS: __________**

**DEMOGRAPHICS OF MIDWIVES**

Age______________Cadre ____________ Work experience___________________ Employment status*: (Full time/Part time)__________________*Hihgest qualification__________________ Marital Status_____________ Clinic__________________________

**SECTION A: FEASIBILITY OF SCREENING FOR DEPRESSION USING SPADe**

In this section you are going to answer questions that were adapted from Structured Assessment of FEasibility (SAFE) Version 1.1 (Bird et al., 2014) to assess the extent to which SPADe is feasible for implementation in antenatal clinics

Please tick the response that applies to you against each question

**BLOCKS SUB-SCALE:** *These items are blocks to implementation.*

| **SN** | **Item** | **Yes** | **Partial** | **No** | **Unable to rate** |
| --- | --- | --- | --- | --- | --- |
| 1 | Do staff require specific training to deliver the SPADe? |  |  |  |  |
| 2 | Is the SPADe complex? |  |  |  |  |
| 3 | Is the SPADe time consuming to provide? |  |  |  |  |
| 4 | Does the SPADe include/require ongoing support and supervision? |  |  |  |  |
| 5 | Does the SPADe require additional human resources? |  |  |  |  |
| 6 | Does the SPADe require additional material resources? |  |  |  |  |
| 7 | Is the SPADe costly? |  |  |  |  |
| 8 | Are there known serious or adverse events associated with the SPADe? |  |  |  |  |

**ENABLERS SUB-SCALE:** *These items are enablers of implementation.*

| SN | Item | Yes | Partial | No | Unable to rate |
| --- | --- | --- | --- | --- | --- |
| 1 | Is the SPADe applicable to the population of interest (e.g. pregnant women using antenatal clinics) |  |  |  |  |
| 2 | Is the SPADe manualised? |  |  |  |  |
| 3 | Is the SPADe flexible (i.e. can it be tailored to the context and situation)? |  |  |  |  |
| 4 | Is the SPADe likely to be effective (i.e. evidence based and expected to produce positive outcomes)? |  |  |  |  |
| 5 | Is the SPADe cost saving? |  |  |  |  |
| 6 | Do the intended goals of the SPADe match the prioritised goals of the Ministry of Health? |  |  |  |  |
| 7 | Can the SPADe be piloted? |  |  |  |  |
| 8 | Is the SPADe reversible? |  |  |  |  |

Adapted from Bird et al. (2014)

**SECTION B: ACCEPTABILITY OF SCREENING FOR DEPRESSION USING SPADe**

In this section you are going to answer questions that were adapted from Acceptability of Decision Rules Instrument (OADRI) (Brehaut et al., 2010) to assess acceptability for implementation of SPADe in antenatal clinics

Please tick the response that applies to you against each question

| **SN** | **Items** | **Strongly disagree** | **Disagree** | **No opinion** | **Don’t Know** | **Agree** | **Strongly agree** |
| --- | --- | --- | --- | --- | --- | --- | --- |
| 1 | SPADe is easy to use |  |  |  |  |  |  |
| 2 | SPADe is easy to remember |  |  |  |  |  |  |
| 3 | SPADe is useful in my practice |  |  |  |  |  |  |
| 4 | Wording of SPADe is clear and unambiguous |  |  |  |  |  |  |
| 5 | My colleagues support use of SPADe |  |  |  |  |  |  |
| 6 | Clients benefit from use of SPADe |  |  |  |  |  |  |
| 7 | SPADe results in improved use of resources |  |  |  |  |  |  |
| 8 | SPADe would increase the chance of lawsuits |  |  |  |  |  |  |
| 9 | Evidence supporting SPADe is flawed |  |  |  |  |  |  |
| 10 | I am already using another protocol or similar strategy |  |  |  |  |  |  |
| 11 | SPADe does not account for important clinical cue |  |  |  |  |  |  |
| 12 | Environment I work in makes it difficult to use SPADe |  |  |  |  |  |  |

Adapted from Brehaut et al. (2010)

**THANK YOU FOR PARTICIPATING IN THIS STUDY**
