# Supplementary material for: Protocol for assessing feasibility, acceptability and fidelity of screening for antenatal depression (FAFSAD) by midwives in Blantyre District, Malawi
Source: Pilot Feasibility Stud. 2021 Jan 26;7:32. doi: 10.1186/s40814-021-00775-6 (PMC7836563; doi:10.1186/s40814-021-00775-6)
Supplement: Supplementary file 5 — Additional file 5. Evaluation of screening for antenatal depression using SPADe among midwives. [file 40814_2021_775_MOESM5_ESM.docx]

**Attachment 5: Evaluation of screening for antenatal depression using SPADe among midwives**

**CODE/INITIALS: __________**

**DEMOGRAPHICS OF MIDWIVES**

Age______________Cadre ____________ Work experience___________________ Employment status*: (Full time/Part time)__________________* Hihgest qualification__________________ Marital Status____________ Clinic_____________________________

**FEASIBILITY OF SCREENING FOR DEPRESSION**

1. Do you feel that SPADe is better able to identify depression than using the usual

questions in Woman’s Health Passport alone? Yes/No

1. Do you feel that the SPADe was well received by pregnant women? Yes/No
2. Do you think pregnant women comfortable with answering the screening questions

for depression? Yes/No

1. Do you feel more confident to identifying depression in pregnant women since you

received training in using SPADe? Yes/No

1. Do you feel more confident in managing depression in pregnant women since you

received training in using SPADe? Yes/No

1. Has the depression screening increased your awareness about the prevalence of

Depression and associated risk factors in antenatal? Yes/No

1. Do you think that the SPADe should be included as a formal part of assessment of

pregnant woman in the Woman’s Health Passport? Yes/No

1. What additional resources or information about antenatal depression should be made available to midwives working in antenatal clinics?

______________________________________________________________________________________________________________________________________________________________________________________________________________________________________________________________________________

1. What else you would like to comment on regarding your experiences either in the training session or using of the SPADe? ______________________________________________________________________________________________________________________________________________________________________________________________________________________________________________________________________________

**ACCEPTABILITY OF SCREENING FOR DEPRESSION**

1. Do you think it is a good idea to screen pregnant women for depression? Yes/No
2. Screening for depression should be routinely conducted on pregnant women Yes/No
3. When you heard that you would be screening pregnant women for depression, what did you think of the idea?

______________________________________________________________________________________________________________________________________________________________________________________________________________________________________________________________________________

1. How comfortable are you with screening for depression using SPADe?

______________________________________________________________________________________________________________________________________________________________________________________________________________________________________________________________________________

1. If you had a choice, would you continue screening for depression during pregnancy using SPADe? Yes/No

14(a) If “Yes”, Why would you continue screening for depression using SPADe?

__________________________________________________________________________________________________________________________________________________________________________________________________________________________________________________________________

14(b) If “No”, Why would you not continue screening for depression using SPADe? __________________________________________________________________________________________________________________________________________________________________________________________________________________________________________________________________

1. What is the most challenging aspect to screening for depression using SPADe?

______________________________________________________________________________________________________________________________________________________________________________________________________________________________________________________________________________

1. Do you have any suggestions that you think might improve the screening process?

______________________________________________________________________________________________________________________________________________________________________________________________________________________________________________________________________________

**FIDELITY OF SCREENING FOR DEPRESSION**

1. Please list out, step by step, how you complete the screening for depression in pregnant women using SPADe (We are interested in your description from the very beginning to the end)

______________________________________________________________________________________________________________________________________________________________________________________________________________________________________________________________________________________________________________________________________________________________________________________________________________________________________________________________________________________________________________________________________________________________________________________________________________________________________________________________________________________________________________________________________________________________________________________________________________________________________________________________________________________________________________________________________________________________________________________________________________________________________________________________________________________________________________________________________________________________________________________________________________________________________________________________________________________________________________________________________________________________________________________________________

1. How do you introduce the screening for depression using SPADe to the pregnant woman?

________________________________________________________________________________________________________________________________________________________________________________________________________________________________________________________________________________________________________________________________________________________________________

1. What have you found works best to get midwives use SPADe to screen depression in pregnant women?

_______________________________________________________________________________________________________________________________________________________________________________________________________________________________________________________________________________________

**THANK YOU FOR PARTICIPATING IN THIS STUDY**
