# Supplementary material for: Protocol for assessing feasibility, acceptability and fidelity of screening for antenatal depression (FAFSAD) by midwives in Blantyre District, Malawi
Source: Pilot Feasibility Stud. 2021 Jan 26;7:32. doi: 10.1186/s40814-021-00775-6 (PMC7836563; doi:10.1186/s40814-021-00775-6)
Supplement: Supplementary file 6 — Additional file 6. Skills acquisition checklist on implementation of SPADe. [file 40814_2021_775_MOESM6_ESM.docx]

**Attachment 6: Skills acquisition checklist on implementation of SPADe**

Instruction: Tick in appropriately in box corresponding to each item

| **SN** | **Items** | **Yes** | **No** |
| --- | --- | --- | --- |
|  | Midwife initially screeen all pregnant women with the 3 Item screener during history taking at booking visit |  |  |
|  | Midwife immediately administers SRQ 20 to all pregnant women who screen positive on the 3 Item screener |  |  |
|  | Midwife immediately refers any pregnant woman who screens positive on SRQ 20 for diagnostic mental health assessment within antenatal clinic |  |  |
|  | Midwife immediately refers to mental health services or within 24 hours any pregnant woman at risk of suicide (who answers ‘Yes’ to questions 16 and 17 on SRQ 20) |  |  |
|  | Midwife actively monitors pregnant women who screen negative on the 3 Item screener or SRQ 20 through ‘watchful waiting’ without any active treatment |  |  |
|  | Midwife provides targeted psychoeducation to all pregnant women who screen positive or negative on the 3 Item screener or SRQ 20 |  |  |
|  | Midwife documents all information regarding pregnant woman’s screening results, diagnosis of depression, medication, and other therapies in the Woman’s Health Passport |  |  |
|  | Midwife continues to liaise closely with mental health services if a pregnant woman is already involved with mental health services |  |  |
|  | Midwife discusses with mental health specialists if pregnant woman is already on antidepressant medication or altenatively refer her to mental health services |  |  |
|  | Mental health specialists assesses and treat pregnant women women with depression in antenatal clinics |  |  |
|  | Mental health specialists refer pregnant women with severe depression and other complex mental disorders to psychiatric unit |  |  |
|  | Mental health specialists provide continuous supportive supervision to midwives |  |  |
|  | Mental health specialists document all information about client condition, treatment and appointments in the Woman’s Health Passport |  |  |
|  | Midwife documents all missed appointments by a pregnant woman and communicate with other services involved in providing support to pregnant woman with or at risk of depression |  |  |
|  | Midwife identifies and addresses issues led to a pregnant woman missing any appointment with antenatal or mental health services |  |  |
|  | Urgently refers to mental health services any pregnant woman whose mental health state is deteriorating |  |  |
